# Selenized milk casein in the diet of BALB/c nude mice reduces growth of intramammary MCF-7 tumors

**DOI:** 10.1186/1471-2407-13-492

**Published:** 2013-10-23

**Authors:** Jenny M Warrington, Julie JM Kim, Priska Stahel, Scott RL Cieslar, Roger A Moorehead, Brenda L Coomber, Milena Corredig, John P Cant

**Affiliations:** 1 Centre for Nutrition Modelling, Department of Animal and Poultry Science, University of Guelph, Guelph, ON N1G 2W1, Canada; 2 Department of Biomedical Sciences, University of Guelph, Guelph, ON N1G 2W1, Canada; 3 Department of Food Science, University of Guelph, Guelph, ON N1G 2W1, Canada

**Keywords:** Selenium, Casein, Mammary tumor, MCF-7 cells

## Abstract

**Background:**

Dietary selenium has the potential to reduce growth of mammary tumors. Increasing the Se content of cows’ milk proteins is a potentially effective means to increase Se intake in humans. We investigate the effects of selenized milk protein on human mammary tumor progression in immunodeficient BALB/c nude mice.

**Methods:**

Four isonitrogenous diets with selenium levels of 0.16, 0.51, 0.85 and 1.15 ppm were formulated by mixing low- and high-selenium milk casein isolates with a rodent premix. MCF-7 cells were inoculated into the mammary fat pad of female BALB/c nude mice implanted with slow-release 17 β-estradiol pellets. Mice with palpable tumors were randomly assigned to one of the four diets for 10 weeks, during which time weekly tumor caliper measurements were conducted. Individual growth curves were fit with the Gompertz equation. Apoptotic cells and Bcl-2, Bax, and Cyclin D1 protein levels in tumors were determined.

**Results:**

There was a linear decrease in mean tumor volume at 70 days with increasing Se intake (P < 0.05), where final tumor volume decreased 35% between 0.16 and 1.15 ppm Se. There was a linear decrease in mean predicted tumor volume at 56, 63 and 70 days, and the number of tumors with a final volume above 500 mm^3^, with increasing Se intake (P < 0.05). This tumor volume effect was associated with a decrease in the proportion of tumors with a maximum growth rate above 0.03 day^-1^. The predicted maximum volume of tumors (V_max_) and the number of tumors with a large V_max_, were not affected by Se-casein. Final tumor mass, Bcl-2, Bax, and Cyclin D1 protein levels in tumors were not significantly affected by Se-casein. There was a significantly higher number of apoptotic cells in high-Se tumors as compared to low-Se tumors.

**Conclusions:**

Taken together, these results suggest that turnover of cells in the tumor, but not its nutrient supply, were affected by dairy Se. We have shown that 1.1 ppm dietary Se from selenized casein can effectively reduce tumor progression in an MCF-7 xenograft breast cancer model. These results show promise for selenized milk protein as an effective supplement during chemotherapy.

## Background

Selenium is an essential trace mineral that is required at greater than 0.15 ppm in the diet of humans and laboratory animals to maximize synthesis of selenoproteins [1]. Se becomes toxic at levels greater than 400 ug /d in the diet and deficient at levels lower than 40 ug/d. Supranutritional supplementation of seleniumat 1 to 4 ppm has shown great promise in cancer prevention [2,3], though the mechanism of the effect remains elusive. Transformed cells are often able to persist and replicate due to a disruption in the regulatory circuitry controlling programmed cell death. Both organic and inorganic forms of Se have been observed to induce apoptosis in several cancer cell lines *in vitro*, including human prostate cancer cells, human leukemia cells, and murine mammary epithelial cells [4-8].

Due to the global variation in soil Se content, there exist Se-deficient populations in the world [9] Further, those populations that receive adequate levels of Se in their diet are likely still below the level of Se required to prevent cancer [10]. Increasing the selenium content of cow’s milk has been suggested to be an effective way of improving selenium intake in humans. The supplementation of Se-yeast in a cow’s diet is the most effective method for increasing milk Se content, where Se is incorporated into milk proteins as selenomethionine during milk synthesis [11]. Consumption of 1 ppm Se from selenized milk protein increased apoptosis and decreased proliferation of chemically induced colon tumors, and decreased the number of mice with tumors 30 weeks after carcinogen exposure [12]. Effects of dairy Se on mammary tumor development has not previously been investigated although organic forms of Se providing 2 ppm dietary Se decreased by 50% the number of chemically induced mammary tumors 22 weeks after carcinogen exposure in rats [6].

The objective of this study was to investigate the effects of selenized milk protein on human mammary tumor progression in immunodeficient BALB/c nude mice. The effects of selenized milk protein on apoptotic circuitry in these human epithelial MCF-7 breast tumor cells were also investigated. We found that each increment in Se intake between 0.16 and 1.15 ppm of diet dry matter caused a decrease in tumor volume after 8 weeks on diets.

## Methods

### Cell culture

Estrogen receptor-positive MCF-7 breast cancer cells were cultured in Eagle’s Minimum Essential Medium (ATCC Catalog No. 30–2003) supplemented with 0.01 mg/mL human insulin and 10% fetal bovine serum. Cells were incubated at 37°C and 5% CO_2_ in air atmosphere. Passage was conducted when cells reached confluency every two to three days. Cells were collected for injection at 80% confluency by centrifuging at 125 x*g* for 5 minutes. Cells were suspended in 50% Matrigel™ Basement Membrane Matrix (BD Biosciences, Mississauga, ON), 50% media at a final concentration of 3 × 10^6^ cells/100 uL.

### Casein isolation and diet formulation

To generate low- and high-Se caseins, Holstein dairy cows were fed 0 or 4.5ppm (dry basis) selenium as Se-yeast (Sel-Plex, Alltech Inc., Kentucky, USA) on top of a basal diet of 0.15 ppm Se from Na_2_SeO_3_. Diets were fed for 3 weeks, after which milk was collected and stored at 4°C until pasteurized and skimmed at 70°C with a flow rate of 1.5L/min. The skim milk was cooled to 45°C and an acid casein precipitate was formed by adding lactic acid (88% food grade) to a pH of 4.6. The casein precipitate was washed twice with cold deionized water, collected on cheese cloth, and drained overnight. The casein was separated into trays and freeze dried at −20°C for 3 days. The product was then ground and stored at 4°C.

Final Se concentrations in the low- and high-Se caseins, measured by fluorometry (AOAC, 2005) were 0.87 and 9.3 ppm, respectively. These low- and high-Se caseins were then sent to Research Diets Incorporated to be mixed with a standard rodent to produce four diets with varying Se levels. AIN-76 diets (Research Diets Inc., New Brunswick, NJ) containing 0.16, 0.51, 0.85, and 1.15 ppm Se (dry basis) were produced by mixing low- and high-Se caseins, to a final concentration of 20% casein in each diet (Table 1). After mixing, diets were gamma-irradiated and stored at 4°C. Proximate nutrient composition of diets was determined at a commercial laboratory according to AOAC (2005) methods.

**Table 1:** Composition of experimental diets

**Ingredient**	**(g/100 g diet)**
Casein	20.0
L-Cystine	0.28
Corn starch	29.9
Maltodextrin	3.3
Sucrose	32.1
Cellulose	4.7
Soybean oil	2.4
Lard	1.9
Mineral mix, no added Se	0.95
Calcium phosphate dibasic	1.23
Calcium carbonate	0.52
Potassium citrate H_2_O	1.56
Vitamin mix	0.95
Choline bitartrate	0.19

### Animal trials

All mice were housed and cared for according to guidelines established by the Animal Care Committeeat the University of Guelph and the Canadian Council on Animal Care. Seventy-two female, athymic, BALB/c nude mice, 6–8 weeks old, were purchased from Charles River Laboratories (Senneville, QC). Mice were housed in autoclaved, ventilated cages and provided with autoclaved water. Water and food were offered *ad libitum*. Food consumption was not measured, however body weights were measured biweekly throughout the trial. They were exposed to a 12-hour light/12-hour dark cycle and fed standard mouse chow (Research Diets, New Brunswick, NJ) once daily at 1800 h. After 1 week of acclimatization, mice were xenotransplanted under isoflurane anaeasthesia with 3 x 10^6^ MCF-7 cells in the mammary fat pad and 90-day release 17β-estradiol pellets (0.5 mg/pellet; Innovative Research of America, Sarasota, FL) subcutaneously between scapulae.

Beginning one week post-surgery, tumor volumes were assessed daily using caliper measurements. Tumor volumes (V) were calculated as V = l × w^2^/2, where l is length and w is width of the tumor. Once tumor volume reached approximately 60 mm^3^ mice were randomized to one of the four treatment diets. Only mice with tumors that reached a palpable volume within 3 weeks after implantation were included in the trial. The animal trial involved 72 mice; 18 mice per diet. 7, 8, 4, and 5 mice from treatments 1, 2, 3, and 4, respectively, were euthanized during this trial. These mice were prematurely euthanized for exhibiting criteria for euthanasia; either clinical signs or because tumor size exceeded 10% of body weight. Each cage of 5 mice was furnished with 2 plastic feeders that were replaced at 1200 h daily with 10 g powdered diet.

After 10 weeks on experimental diets, mice were sacrificed by CO_2_ asphyxiation, and tumors and livers were excised, weighed and frozen in liquid nitrogen prior to storage at −80°C. A section of tumor was also stored in 10% formalin solution for at least 24 hours, after which it was embedded in paraffin wax.

### Western blotting

Western blots were performed on tumor tissue from each subject to measure levels of cyclin D1, Bcl-2, and Bax. Tumor tissues were homogenized in 2 μL RIPA lysis buffer/mg tissue, containing 10 μL/mL protease inhibitor. Protein concentrations in tissue homogenates were measured according to Bradford (1976) with bovine serum albumin as the standard. Membranes were blocked for one hour with 5% (wt/vol) skim milk at 4°C and incubated with rabbit anti-cyclin D1 (1:1000 dilution, Cell Signaling, Danvers, MA), rabbit anti-Bcl-2 (1:1000 dilution, Cell Signaling, Danvers, MA), rabbit anti-Bax (1:1000 dilution, Cell Signaling, Danvers, MA) or rabbit anti-β-tubulin (1:1000 dilution, Cell Signaling, Danvers, MA) on a rocking platform. After washing with TBST (TBS, 1% (vol/vol) Tween 20), membranes were incubated with peroxidase-conjugated anti-rabbit secondary antibody (1:2000 dilution, Cell Signaling, Danvers, MA) for 1 hour at room temperature on a rocking platform. Immunoreactive proteins were visualized by chemiluminescence (ECL Western Blotting Detection Reagents) using horseradish peroxidase-linked secondary antibody (anti-rabbit immunoglobulin, 1:2000 dilution). β-tubulin was used to normalize protein loads between blots.

### Detection of apoptosis

Wax-embedded tissues from the 4 largest tumors on each treatment were sectioned at 5 μm onto polarized slides for colorimetric TUNEL assay. To visualize and quantify tumor cell death, TUNEL assay was performed using an *in situ* Cell Death Detection Kit (Promega, Madison, WI, USA) according to the manufacturer’s protocol. Nuclei were counterstained using Harris-modified hematoxylin, and slides were mounted. The number of apoptotic cells was expressed as a percentage of total cells, counting 1500–2000 nuclei per sample.

### Se Status

Tumor samples were freeze-dried and digested in nitric acid in a 90°C sand bath. The digestate was then brought up to volume (10mL) and analyzed using Inductively Coupled Plasma Mass Spectroscopy (AOAC, 2005). Selenium values are reported on a dry matter basis.

### Modeling

To compare tumor growth characteristics across treatments, trajectories of individual tumor volume (V_t_) versus time (t) were fitted with the Gompertz equation,

(1) $$V_{t}=V_{\max}e^{‒be^{‒\mathrm{ct}}}$$

where V_max_ is the asymptotic upper bound that the tumor volume approaches as time approaches infinity, and b and c describe the growth. The maximum specific growth rate (c) occurs at the inflection point, t_inflection_ = ln(b)/c. Best estimates for parameters V_max_, b and c were obtained by minimizing residual sums of squares between predicted and observed tumor volumes using Excel Solver. The parameter estimates were then used to generate predicted volumes at weekly intervals after assignment to diet. Goodness of fit to each curve was assessed by root mean square prediction error (rMSPE), as a percentage of the mean observed tumor volume, calculated as:

(2) rMSPE%=∑i=1npredi−obsi2n∑i=1nobsin

where pred_i_ is the i-th prediction, obs_i_ is the i-th observation, and n is the number of observations.

Specific growth rate (a) at any point in time can be calculated using the following:

(3) $$a=\mathrm{bc}e^{‒\mathrm{ct}}$$

The specific growth rate at 70 d of dietary treatment was calculated as:

(4) $$a_{\mathrm{final}}=\mathrm{bc}e^{‒c70}$$

### Statistical analysis

**Figure 1:**
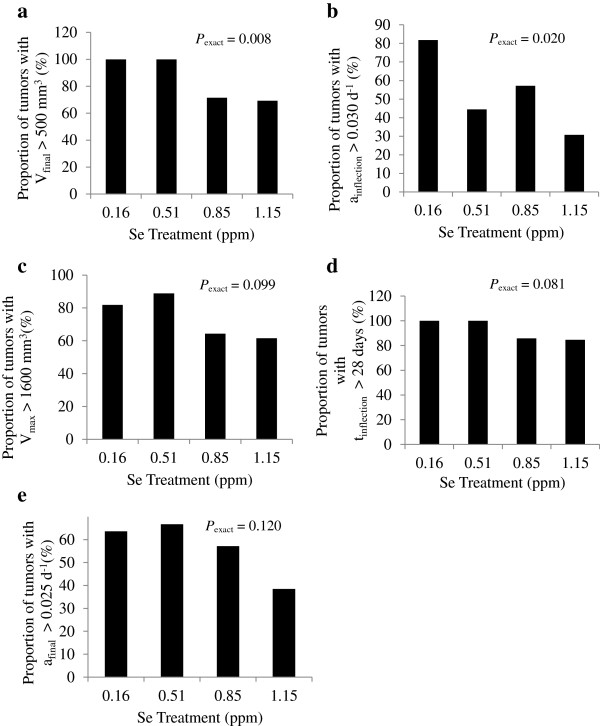
**Proportion of large and fast-growing tumors within each treatment group.** Proportion of tumors within each Se-casein treatment with **a)** final volume above 500 mm^3^, **b)** maximum growth rate above 0.03 d^-1^, **c)** maximum volume (V_max_) above 1600 mm^3^, **d)** inflection point after 28 days, and **e)** final growth rate above 0.025 d^-1^ were subjected to an exact Cochran-Armitage test for linear effect of dietary Se level. *P*-values are shown as *P*_exact_.

The general linear models procedure of SAS was used to detect differences in observations between treatments by one-way analysis of variance. To eliminate discrepancies in initial growth rates, mice put on treatment three weeks or longer post-inoculation were excluded from the dataset. Tumors were also excluded based on a confidence grade, where a grade of 1 was assigned to regular-shaped tumors, and a grade of 0 to those with an irregular shape for which volume could not be accurately measured. Tumors with a grade of 0 were then excluded from analysis. Tumors were also excluded if their final volume was below 30 mm^3^, indicating inadequate estrogen implantation. Number of tumors excluded from 0.16, 0.51, 0.85, and 1.15 ppm treatments were 3, 3, 1, and 1, respectively. Orthogonal linear and quadratic contrasts of dietary Se level were determined with coefficients calculated in PROC IML of SAS to match the measured Se level. Because of a large variation around mean tumor growth characteristics within treatments, the proportion of tumors with growth characteristics above or below specified threshold values were calculated for each treatment (see Figure 1). Characteristics considered were final observed tumor volume (V_final_), c, V_max_, t_inflection_, and a_final_. The maximum specific growth rate (c) may also be described as the specific growth rate at the inflection point (a_inflection_). Linear effects of dietary Se level on the proportions were detected using an exact Cochran-Armitage trend test in PROC FREQ of SAS. *P*-values less than 0.05 were reported as significant.

## Results

### Tumor growth dynamics

**Figure 2:**
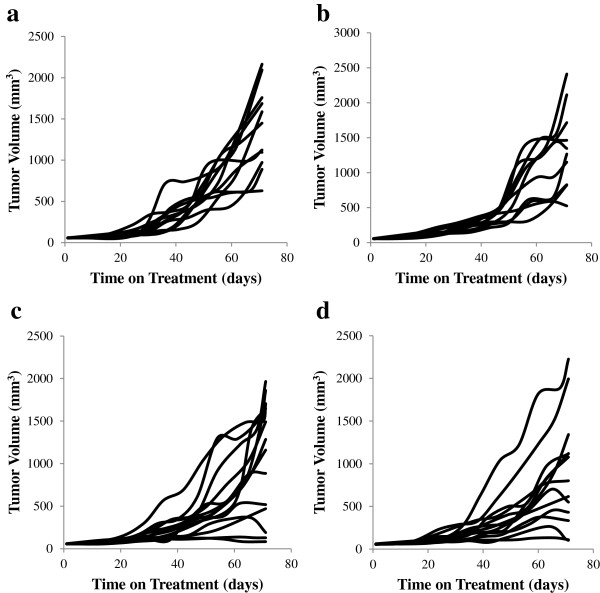
**Tumor volume growth curves for individual mice in each treatment group.** MCF-7 cells were xenografted into mammary fat pads of nude BALB/c mice implanted with slow-release estrogen pellets. Once tumor volumes reached 60 mm^3^ in volume, mice were assigned to dietary treatments of Se-casein at **a)** 0.16 ppm Se, **b)** 0.51 ppm Se, **c)** 0.85 ppm Se, and **d)** 1.15 ppm Se. Tumor volumes were estimated from caliper measurements once per week during treatment.

**Figure 3:**
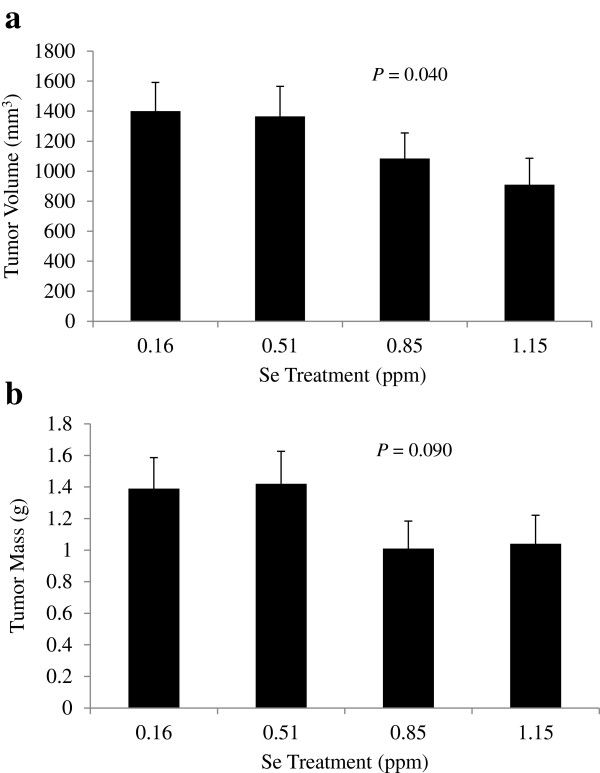
**Final tumor volumes and masses. a)** volume estimated from caliper measurements and **b)** mass measured after tumor excision, on day 70 of Se-casein treatment. Values are means ± SE for each dietary treatment group (n = 11, 10, 14 and 13, respectively). *P*-values represent linear effects of dietary Se level.

**Figure 4:**
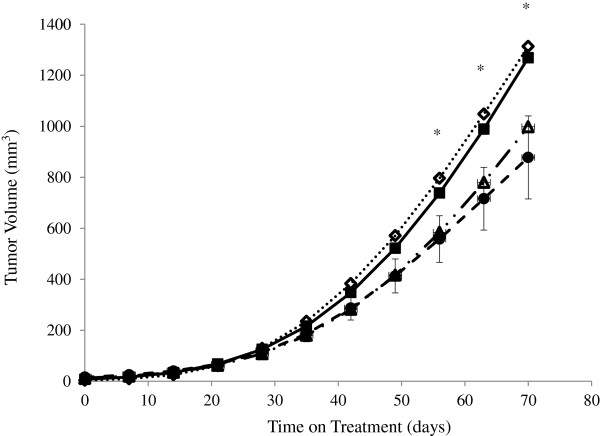
**Mean tumor volumes predicted from fits of Gompertz equation to individual growth curves.** Values are means for each Se-casein treatment (n = 11, 9, 14 and 13, respectively). Error bars represent pooled SE of the mean. Asterisks indicate significant linear effects of dietary Se from Se-casein (*P*< 0.05). 0.16 ppm Se (n=11), 0.51 ppm Se (n=9), 0.85 ppm Se (n=14), 1.15 ppm Se (n=13).

Growth of mammary tumor volumes during the 10 weeks of dietary treatment exhibited exponential growth to a plateau (Figure 2). There was a linear decrease in mean tumor volume at 70 days with increasing Se intake (*P* = 0.040; Figure 3a) and a tendency for final tumor mass to decrease (*P* = 0.090; Figure 3b). Final tumor volume decreased 35% between 0.16 and 1.15 ppm Se. Within each treatment, some tumors reached their maximum volume by 70 days, while others were still in a quasi-exponential growth phase at 70 days. In addition to variation in the time at which plateau was reached, there was a large variability in the plateau volume itself. Chignola et al. [13] speculated that this growth variability is an intrinsic property of each individual tumor. Even multicellular tumor spheroids grown under controlled culture conditions *in vitro* exhibit large growth variability [13]. In order to account for these variables, growth curves were fit with the Gompertz equation which generates estimates of the plateau volume (V_max_), and volumes (Equation 1) and specific growth rates (Equation 3) at any time point. Root MSPE averaged 16% of mean tumor volume with no difference between treatments. One mouse on 0.51 ppm Se was excluded from Gompertz analysis due to a physiologically implausible best-fit b-value > 100,000. There was no effect of Se-casein on mean V_max_ or other Gompertzian growth parameters of the mammary tumors (Table 2). Using the fitted Gompertz parameters, tumor volumes were predicted at weekly intervals and averaged by treatment to generate tumor growth curves for each Se inclusion level (Figure 4). Mean volumes at days 56, 63 and 70 on diet were significantly different between treatments, with decreasing volumes as Se-casein intake increased.

**Table 2:** Gompertz fits to tumor growth curves and parameter estimates.

	**Dietary Se level (μg/g dry matter)**					
Measurement	0.16	0.51	0.85	1.15	SE	*P*^1^
n	11	9	14	13		
rMSPE (% of mean)	14	16	17	16	3	0.458
V_max_ (mm^3^)^2^	3451	5739	3486	2708	1162	0.348
b^3^	9.76	7.91	8.24	6.72	1.5	0.139
c (day^-1^)^4^	0.039	0.029	0.042	0.027	0.006	0.368
t_inflection_ (day)^5^	62	76	59	66	10	0.894
a_final_ (day^-1^)^6^	0.026	0.026	0.025	0.023	0.005	0.582

^1^Linear effect of dietary Se level.

^2^Maximum volume.

^3^Gompertz growth parameter.

^4^Gompertz growth parameter describing maximum specific growth rate.

^5^Time of inflection point describing time at which maximum growth rate occurs.

^6^Final specific growth rate at day 70 of dietary treatment.

Effects of Se-casein on tumor growth were also evaluated by counting the number of large and fast-growing tumors on each treatment. The proportion of tumors with a final volume above 500 mm^3^ (Figure 1a) and the proportion of tumors with a maximum growth rate above 0.03 d^-1^ (Figure 1b) were both linearly decreased by Se inclusion level (*P*_exact_< 0.02). Proportions of tumors with a large V_max_, t_inflection_, or specific growth rate at 70 days were not affected by treatment (Figure 1c - e).

### Tissue analysis

**Figure 5:**
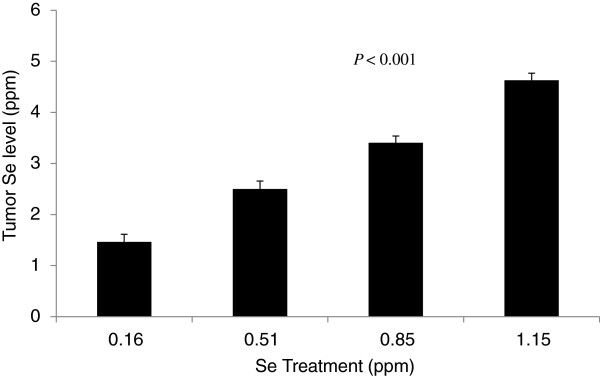
**Tumor Se levels on a dry matter basis.** MCF-7 tumors were excised from mice at day 70 of Se-casein treatment and subjected to Se analysis. Values are means ± SE for each treatment group (n = 11, 10, 14, and 13, respectively). The *P*-value represents the linear effect of dietary Se level.

**Figure 6:**
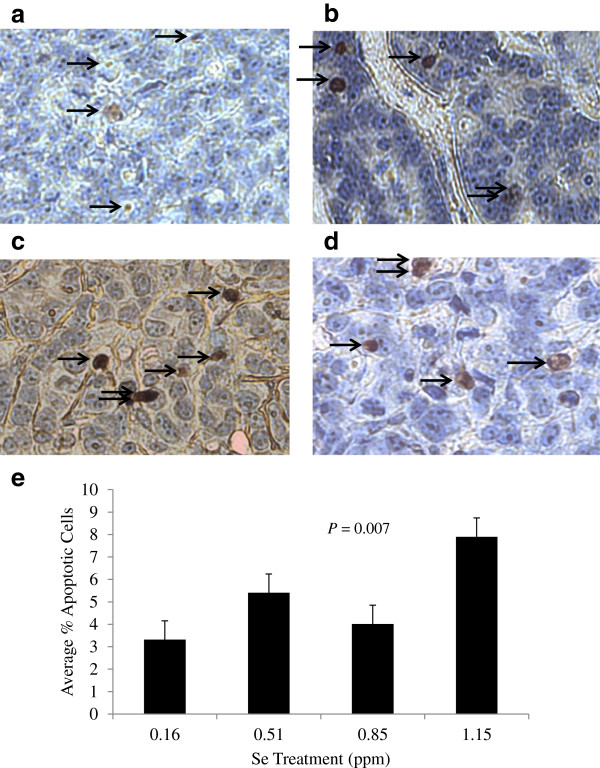
**Effect of Se-casein on apoptotic cell number.** After 70 d on Se-casein treatment, the 4 largest MCF-7 tumors on each treatment were excised, embedded in wax, and sectioned at 5 μm onto microscope slides.The TUNEL assay was used to identify apoptotic cells and nuclei were counterstained with hematoxylin. **a)** mouse 13, 0.16 ppm Se. **b)** mouse 26, 0.51 ppm Se. **c)** mouse 3, 0.85 ppm Se. **d)** mouse 15. 1.15 ppm Se. Arrows indicate apoptotic cells. **e)** Apoptotic cell number was expressed as a percentage of total nuclei, counting 1500–2000 nuclei per sample. Values are means ± SE for each treatment group. The *P*-value represents the linear effect of dietary Se level.

Average Se content of tumors increased linearly with Se intake from Se-casein (*P*< 0.001; Figure 5). The proportion of apoptotic cells in the 4 largest tumors on each treatment, which was assayed by DNA nick-end labeling (Figure 6a - d), also increased 2.4-fold with increasing Se levels (*P* = 0.007; Figure 6e).

**Figure 7:**
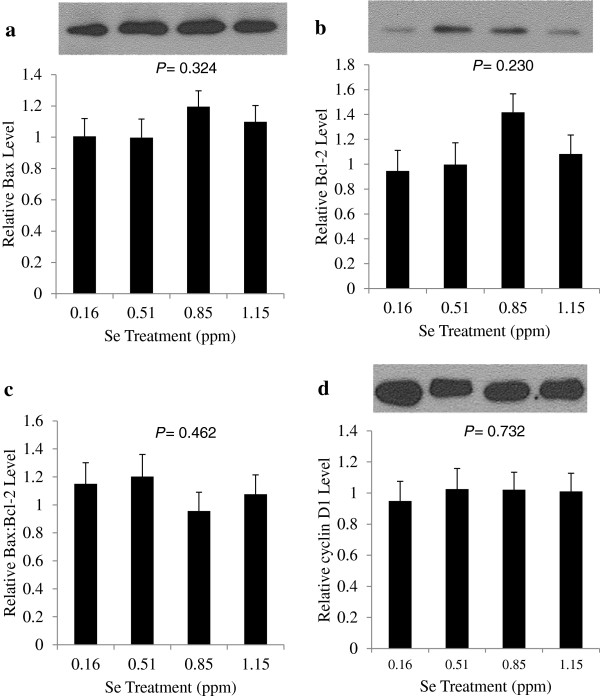
**Effects of selenium treatments on Bax, Bcl-2 and cyclin D1 protein levels.** After 70 d of Se-casein treatment, tumors were excised from mice and subjected to western blot analysis for **a)** Bax, **b)** Bcl-2, **c)** Bax:Bcl-2 ratio and **d)** cyclin D1. Expression levels were normalized to β-tubulin. Values in bar graphs are means ± SE for each treatment group (n = 11, 10, 14 and 13, respectively). *P*-values represent linear effects of dietary Se level.

The expression of Bcl-2 protects cells against apoptosis, while Bax opposes the action of Bcl-2 and aids in the induction of apoptosis. The ratio of Bax to Bcl-2 is often considered a rheostat to control the level of apoptosis occurring in tissue. Western blot analysis of tumor tissue showed no significant treatment effects on the expression of Bcl-2 or Bax proteins, or the ratio of Bax:Bcl-2 (Figure 7a - c).

The level of cyclin D1 expression was chosen as a marker of cell proliferation, as it is commonly over-expressed in breast cancer cells [14,15]. Western blot analysis of tumor tissue from each mouse subject did not reveal a significant difference in cyclin D1 expression between treatments (Figure 7d).

## Discussion

Our findings show, for the first time, that dietary Se is effective at reducing growth of human mammary tumors *in situ*. The tumors originated from MCF-7 cells implanted in the mammary stroma of immune-compromised mice. The dietary Se was provided in casein isolated from the milk of cows fed Se-yeast in their diet. Increasing dietary Se from 0.16 to 1.15 ppm caused a linear decrease in tumor volumes and the number of large tumors after 8 weeks (tumors with Vfinal > 500 mm^3^). The tumor volume effect was associated with a decrease in the number of tumors with a fast maximum growth rate. Although these observations indicate that growth rate was reduced, the maximum volume that tumors were predicted to reach (V_max_), and the number of tumors with a large V_max_, were not affected by Se-casein. Maximum volume and growth rate were generated using Gompertz fits of the observed growth data. Growth rate of tumors is related to the net difference between proliferation and death of individual cells, while final volume is related to the ability to maintain oxygen and nutrient supply to the tumor, particularly its inner core [16]. Thus, Se-casein appears to affect cancer cell turnover but not supply or extraction of nutrients by the tumor.

Our results add a new dimension to the findings that dietary Se prevents oncogenesis in chemical-induction models of mammary and colorectal cancer [6,12], and to the growing body of evidence that Se compounds are effective against established tumors [17]. Selenium is thought to affect tumor growth by inducing cell cycle arrest and apoptosis [18-20]. Generally speaking, inorganic selenium is reduced to hydrogen selenide which results in reactive oxygen species-mediated induction of single-strand DNA breaks and apoptosis in various cancer cell lines, including leukemia, mammary and prostate cancers [21-23]. Organic selenium sources, on the other hand, are able to induce apoptosis without the genotoxic effects, ostensibly via the metabolite methylselenol [6]. Putative mechanisms by which methylselenol prevents cancer include caspase activation and dephosphorylation of pro-survival Akt and extracellular signal-related kinase 1/2 [24].

Casein isolated from the milk of cows fed Se-yeast contains organic Se, primarily in the forms of selenomethionine and selenocysteine [11]. These organic forms of Se are readily concentrated in tissues because of sequestration in proteins. In the current study, tumors from mice fed 1.15 ppm Se-casein accumulated an average Se concentration of 4.58 ppm, whereas s.c. injection of 1.5 ppm inorganic Se as selenite in a previous study resulted in MCF-7 tumor Se concentrations of 1.55 ppm [25]. Whether the higher tumor Se concentration due to Se-casein consumption translates into greater exposure to hydrogen selenide or methylselenol remains unknown. However, we observed a significantly higher number of apoptotic cells in high-Se tumors as compared to low-Se tumors. *In vitro*, the IC_50_ of SeMet against MCF-7 cells was 45 μM [26]. Assuming it is all SeMet, the 4.58 ppm Se we found in mammary tumors is equivalent to a concentration of 23 μM SeMet. The 35% decrease in final tumor volume we observed on the high-Se diet is consistent with tumor Se concentrations slightly less than the IC_50_. The pro-apoptotic effect of Se on cancer cells *in vitro* and *in vivo* is well documented [5,6,17] but effects of dietary Se on apoptosis in human mammary tumors have not previously been reported.

Redman and coworkers [26] investigated the effects of SeMet on four cell lines *in vitro*: MCF-7 breast carcinoma, UACC-375 melanoma, DU-145 prostate cancer, as well as normal diploid fibroblasts. This study investigated the IC_50_ of SeMet for for each cell line. SeMet concentrations ranged from 100–10000 μM. SeMet inhibited growth in all cell lines in a dose-dependent manner. In MCF-7 cells, cell viability was not affected by 0.01-10 μM, while 100–1000 μM significantly inhibited cell growth. In UACC-375 melanoma cells, concentrations greater than 1 μM were required to significantly inhibit cell growth. In prostate cancer cells DU-145, concentrations beyond 10 μM showed a marked decline in cell growth. In contrast to the micromolar concentrations of SeMet shown to inhibit cancer cell lines, inhibition of growth in diploid fibroblasts required millimolar concentrations. These results indicated that DU-145 prostate cancer cells are the most sensitive to SeMet treatment with a IC_50_ of 40 μM, followed by MCF-7 and UACC-375 with 45 μM and 50 μM, respectively. Fibroblasts required 1 mM SeMet to induce 50% inhibition. According to these results, cancer cells may be more sensitive to selenium treatment than normal cells [26]. It was postulated that these discrepancies may be due to differences in uptake and metabolism of SeMet to anticarcinogenic metabolites, as SeMet may be metabolized to methylselenol or SeCys, which in turn is hydrolyzed to hydrogen selenide [26].

Similar to Kaeck et al. [27], we found no effect of Se on Bax or Bcl-2 expression in tumors, despite an increase in apoptosis. In contrast, MSeA increased Bax and decreased Bcl-2 expression in three lines of prostate cancer cells [28]. While Bcl-2 and Bax are considered the main players in controlling programmed cell death, apoptosis is an intricate process with several points of control that have been shown to be affected by Se, including expression of Bcl-x1, Bak and Bid [28,29]. The results herein indicate that Se-casein was able to induce apoptosis in MCF-7 cells independently of the Bax:Bcl-2 rheostat, suggesting an alternative apoptotic pathway is being targeted.

In addition to apoptosis, tumor growth is determined by cell proliferation. Se is known to downregulate several genes controlling the expression of cell cycle proteins, including cyclins A and cyclin D1 [20]. Cyclin D1 is an important regulator in the early stages of the cell cycle, controlling the transition from the first gap phase to the synthesis phase. We chose cyclin D1 as a marker of tumor proliferation. While this protein is overexpressed in over half of breast cancer cases,cyclin D1 levels in MCF-7 cells are similar to those found in normal mammary epithelial cells [14,15,30], yet cyclin D1 has been shown to play an essential role in cell cycle progression in MCF-7 cells [31]. We observed no effect of Se-casein treatment on cyclin D1 expression. *In vitro* study shows that 5 μM MSeA downregulates cyclin D1 in premalignant human breast cells at an early time-point and upregulates cyclin D1 at a later time point [32]. Thus, interruption of cyclin D1-mediated cell cycle progression does not appear to be responsible for the inhibitory effects of Se-casein on tumor growth observed in the current study. Selenium, however, has been shown to downregulate several genes controlling the expression of cell cycle proteins, including cyclin A, CDC25A, CDK4, PCNA and E2F [33].

Many different forms of dietary Se have been tested for efficacy against the development and progression of cancer including SeMet, MSeA, SeMSC (Se-methylselenocysteine), and Se-enriched broccoli, garlic and milk protein [6,12,27,34,35]. The highest dose of Se we administered via dietary casein was 1.15 ppm, which was insufficient to cause more than a 35% decrease in tumor growth. However, the linearity of the response to dose suggests that higher doses could have a greater inhibitory effect. Organic Se doses up to 5 ppm Se have been fed to animals in studies of cancer chemoprevention. Another option might be to use Se-casein in conjunction with other chemotherapeutics. Se appears to sensitize cancer cells to apoptosis while reducing the toxic effects of therapy and selectively protecting normal cells [36]. For instance, Li et al. [37] showed that Se in the form of MSeA sensitized MCF-7 cells to doxorubicin-induced apoptosis [37]. Another study found that MSeA acted synergistically with paclitaxel in the treatment of triple-negative breast cancer to increase induction of caspase-mediated apoptosis, cell cycle arrest, and inhibition of cell proliferation [38].

We have shown that 1.15 ppm dietary Se from selenized casein can effectively reduce tumor progression in an MCF-7 xenograft breast cancer model. Results of a DNA nick-end labeling assay support the claim that Se-casein reduces breast cancer cell growth by increasing the number of cells undergoing apoptosis.The literature indicates that the optimal Se intake is 250 – 300 ug per day, however the interaction of Se with other elements must be considered [39]. These elements include, but are not limited to, As, Cu, Ni, Co, Cr, Mn, Zn, Cd, Sn, Pb, Hg, Bi, Mo, Ag, and Au, Evidence from animal experiments suggest that chronic exposure to these elements counteracts the anticarcinogenic effects of Se [39]. This indicates that the presence of these elements in the diet must be well characterized before a claim can be made regarding Se-casein as a cancer-protective supplement.

While the average Se intake in most countries is sufficient to prevent Se deficiencies, it may be suboptimal for protection against a number of adverse health conditions. This is because the amount of selenium in the human diet is largely dependent on the soil content where crops destined for human consumption are cultivated. Therefore, providing Se-enriched casein through milk has the potential to not only prevent deficiency, but also provide the supranutritional levels required to prevent a disease like cancer. This study showed the potential for Se-casein to be an effective treatment of breast cancer, suggesting its potential role in adjuvant therapy. Further study is required to elucidate the precise mechanism through which supranutritional Se-casein levels reduce carcinogenesis. The effects of high-Se casein on normal cells in addition to cancerous cells should also be well-characterized before it may be approved as an effective dietary supplement for chemoprevention in order to eliminate safety concerns.

## Conclusions

Increasing dietary Se from 0.16 to 1.15 ppm caused a linear decrease in tumor volumes and the number of large tumors after 8 weeks. The tumor volume effect was associated with a decrease in the number of tumors with a fast maximum growth rate, however the maximum volume that tumors were predicted to reach and the number of tumors with a large maximum volume were not affected by Se-casein. Taken together, this suggests that dairy Se affects the turnover of cells in the tumor, but not its nutrient supply. We have shown that 1.1 ppm dietary Se from selenized casein can effectively reduce tumor progression in an MCF-7 xenograft breast cancer model. These results show promise for selenized milk protein as an effective supplement during the course chemotherapy.

## Abbreviations

Akt/PKB: Protein Kinase B; Bak: Bcl-2 homologous antagonist killer; Bax: Bcl-2 associated X protein; Bcl-2: B-cell lymphoma 2; DAB: Diaminbenzidine; IC50: Half maximal inhibitory concentration; MSeA: Methylseleninic acid; SeMet: Selenomethionine; TUNEL: Terminal deoxynucleotidyltransferasedUTPnick end labeling.

## Competing interests

The authors declare that they have no competing interests.

## Authors’ contributions

JW cultured cells, formulated diets, conducted the animal trial, collected and analyzed samples, ran statistical analyses, and drafted the manuscript. JK participated in tissue analysis and preparation for publication of the manuscript. PS and SC participated in milk processing and diet formulation. BC participated in the conception, design, and drafting of the manuscript. RM participated in design and drafting of the manuscript. MC participated in conception and design of the manuscript. JC participated in conception, design, statistical evaluation and interpretation of the results, and provided significant input into drafting of the manuscript. All authors have read and approved the manuscript for publication.

## Pre-publication history

The pre-publication history for this paper can be accessed here:

http://www.biomedcentral.com/1471-2407/13/492/prepub
